# Medical priority dispatch codes—comparison with National Early Warning Score

**DOI:** 10.1186/s13049-016-0336-y

**Published:** 2016-12-03

**Authors:** Marko Hoikka, Sami Länkimäki, Tom Silfvast, Tero I. Ala-Kokko

**Affiliations:** 1 Medical Research Centre, Research Unit of Surgery, Anaesthesia and Intensive Care and Department of Anaesthesiology, Division of Intensive Care, University of Oulu and Oulu University Hospital, PO BOX 21, , FI-90029 Oulu, OYS Finland; 2 Emergency Medical Service, Department of Emergency Medicine, Helsinki University Central Hospital and University of Helsinki, FI-00029 Helsinki, HUS Finland

**Keywords:** Emergency medical services, Medical dispatch codes, Early warning score, Triage

## Abstract

**Background:**

In Finland, calls for emergency medical services are prioritized by educated non-medical personnel into four categories—from A (highest risk) to D (lowest risk)—following a criteria-based national dispatch protocol. Discrepancies in triage may result in risk overestimation, leading to inappropriate use of emergency medical services units and to risk underestimation that can negatively impact patient outcome. To evaluate dispatch protocol accuracy, we assessed association between priority assigned at dispatch and the patient’s condition assessed by emergency medical services on the scene using an early warning risk assessment tool.

**Methods:**

Using medical charts, clinical variables were prospectively recorded and evaluated for all emergency medical services missions in two hospital districts in Northern Finland during 1.1.2014–30.6.2014. Risk assessment was then re-categorized as low, medium, or high by calculating the National Early Warning Score (NEWS) based on the patients’ clinical variables measured at the scene.

**Results:**

A total of 12,729 emergency medical services missions were evaluated, of which 616 (4.8%) were prioritized as A, 3193 (25.1%) as B, 5637 (44.3%) as C, and 3283 (25.8%) as D. Overall, 67.5% of the dispatch missions were correctly estimated according to NEWS. Of the highest dispatch priority missions A and B, 76.9 and 78.3%, respectively, were overestimated. Of the low urgency missions (C and D), 10.7% were underestimated; 32.0% of the patients who were assigned NEWS indicating high risk had initially been classified as low urgency C or D priorities at the dispatch.

**Discussion and conclusion:**

The present results show that the current Finnish medical dispatch protocol is suboptimal and needs to be further developed. A substantial proportion of EMS missions assessed as highest priority were categorized as lower risk according to the NEWS determined at the scene, indicating over-triage with the protocol. On the other hand, only a quarter of the high risk NEWS patients were classified as the highest priority at dispatch, indicating considerable under-triage with the protocol.

**Electronic supplementary material:**

The online version of this article (doi:10.1186/s13049-016-0336-y) contains supplementary material, which is available to authorized users.

## Background

Emergency medical dispatching plays an important role in the chain of medical care and patient survival [1]. Critical components of efficient emergency medical dispatching include correct risk assessment and emergency patient identification, management of available emergency medical service (EMS) resources, and maintaining an appropriate call processing time in life-threatening emergencies. Over-triage of calls leads to inappropriate use and overload of EMS units, whereas under-triage may negatively impact patient survival [2]. Although there are no standards for evaluating dispatcher performance accuracy, studies have shown substantial discrepancies in priority assessment between dispatch centres and EMS personnel on the scene [3, 4]. Different countries show substantial variation with regard to dispatch protocols and organization of emergency medical dispatch systems [5].

Compared to most other scoring systems, the National Early Warning Score (NEWS) has shown better performance for medical risk assessment in the hospital setting [6]. Thus, use of the NEWS has been implemented in several hospitals in Finland, but not yet by EMSs. Some previous findings demonstrate that elevated NEWS among unselected prehospital patients is associated with an increased risk of adverse outcomes, suggesting that NEWS may also be useful in the prehospital setting [7]. Accordingly, the Royal College of Physicians recommends the use of the NEWS to standardize the assessment of acute illness severity throughout the entire chain of medical care, including in the prehospital phase [8].

There is a lack of data regarding the efficiency of the dispatch protocol. To try to fill this gap we wanted to assess the usability of the NEWS in this context. We hypothesized that the priority dispatching protocol currently used in Finland is suboptimal and would overestimate and underestimate the patients’ medical risk. The aim of this study was to evaluate the accuracy of the protocol by comparing risk assessment guided by the national criteria-based dispatch protocol at the time of the emergency call with the NEWS at the EMS scene to quantify the rate of over- and under-triage.

## Methods

This study was performed in two hospital districts—Kainuu and Länsi-Pohja—in northern Finland, comprising mostly suburban and rural settings. These areas are home to a total of 140,000 inhabitants, representing 2.6% of the Finnish population, with a population density of 4.7 inhabitants per square kilometre. Both areas are covered by the same emergency medical communication centre (EMCC). The hospital districts organize the emergency medical services, which respond to a total of 35,000 emergency missions annually.

Finland has a national dispatch authority with six regional EMCCs. These centres organize the responses to medical calls and calls for fire and rescue services and police, all of which are accessed through the common European emergency phone number 112. Emergency dispatchers undergo a national 18-month formalized training. They are responsible for evaluating all incoming emergency phone calls and making risk assessment following a criteria-based national standardized dispatch protocol. For medical calls, risk assessment is based on the severity of the patient’s main complaint, clinical condition, and the mechanism of injury. In such calls, the dispatcher’s initial focus is to exclude the possibility of cardiac arrest, and then to identify the most appropriate keyword to describe the reason for the call. The national emergency medical dispatch protocol includes 40 medical keywords—such as chest pain, falls, and seizure—as well as several rescue service and police tasks to which EMS responses can be added. Each keyword carries predetermined criteria questions that help the dispatcher to determine the urgency of the call. Calls are prioritized into four categories, from A (highest priority) to D (lowest priority), and the code given to the EMS units consists of two parts: keyword and priority. The Ministry of Social Affairs and Health is responsible for developing and updating the dispatch protocol used for medical emergencies.

Priority code A is used if the patient has a presumable or evident life-threatening disturbance of vital functions (respiration, circulation, or consciousness) or if there is a high-energy mechanism of injury (Table 1). Priority code B is used if there is a possible threat of vital function failure or if the mechanism of injury seems likely to lead to it. Both A and B priority codes are urgent, indicating that the call should immediately be transmitted to the closest EMS unit(s). In A priority responses, a prehospital physician unit is included, if available. The low urgency C and D priority codes are used if life-threatening signs or symptoms are confidently excluded. Priority code C is assigned if the patient has only minor symptoms or there is a low-energy mechanism of injury. Priority code D is assigned if the patient is stable but needs to be assessed by an EMS unit.

**Table 1:** Priority definitions and suggested responses in the Finnish EMS system

Priority code	A	B	C	D
Definitions	Serious disturbance of vital functions	Suspicion of failure of vital functions	Minor symptoms	No disturbance of vital functions
	High-energy mechanism of injury	Mechanism of injury is suspected to lead on failure of vital functions	Low-energy mechanism of injury	
Dispatch priority	Immediately	Immediately	Patient reached within 30 min	Patient reached within 120 min
EMS unit response	FRU + ALS + (HEMS)	ALS (+ FRU if quicker on scene)	BLS or ALS	BLS
	Lights and sirens	Lights and sirens	Normal driving	Normal driving

*FRU* first responding unit, *ALS* advanced life support unit, *BLS* basic life support unit, *HEMS* Physician helicopter unit

The National Early Warning Score (NEWS) is a standardized tool for evaluating medical risk [8]. In the NEWS system, a score of 0–3 is allocated to each of six physiological measurements: respiratory rate, oxygen saturation, temperature, systolic blood pressure, heart rate, and level of consciousness. The magnitude of the score reflects how much the parameter deviates from normal. The score is then aggregated, and a weighting score of two is added if the patient receives supplemental oxygen. Based on the total score, the medical risk can be categorized into three groups: low (NEWS, 0–4), medium (NEWS, 5–6 or any individual physiological parameter score of 3), and high (NEWS ≥7) (See Additional file 1).

**Fig. 1:**
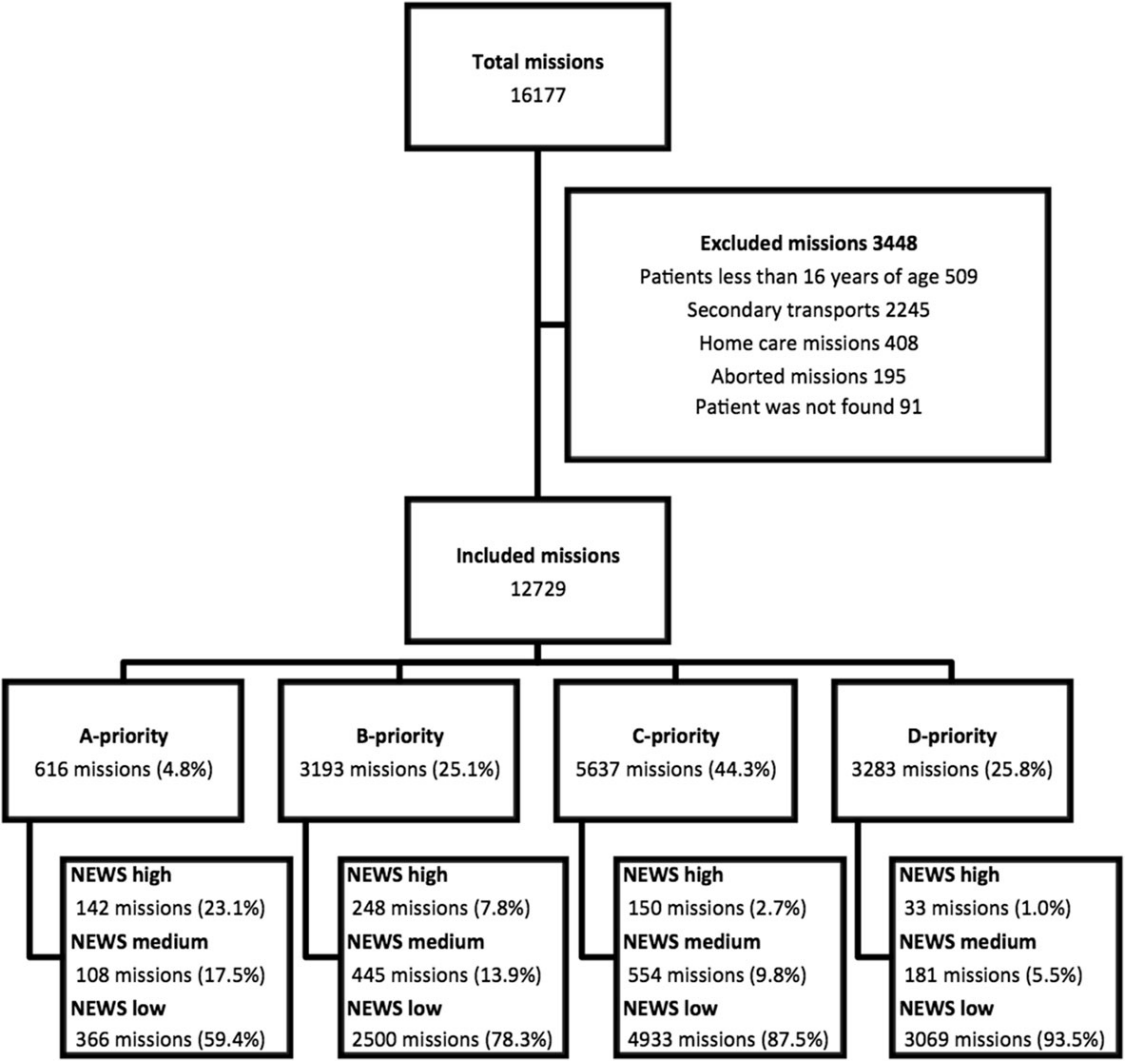
Flow chart of study cohort, and mission distribution according to priorities and National Early Warning Score (NEWS)

EMS providers prospectively recorded data for the present study on EMS charts between 1.1.2014 and 30.6.2014. In the Kainuu region, the main author manually transferred the data from the paper EMS charts to the statistical program. In the Länsi-Pohja region, the data were electronically transferred from the EMS database (Merlot Medi®, CGI). The transferred data included only objective values, so the subjective bias was minimal during the data collection. Unclear markings (eg, poor handwriting) were excluded from the data. The study included all EMS missions with patient contact within every priority category. Secondary (inter-facility) transports and missions where patients were not encountered (aborted mission or patient not found) were excluded. Missions involving patients less than 16 years of age were excluded because the NEWS has not been validated in children (Fig. 1). Collected data included priority and dispatch code, demographic data, and the first clinical variables measured on the scene (systolic blood pressure, heart rate, respiratory rate, Glasgow coma score, oxygen saturation, and temperature). The calculations of the NEWS for each patient were made automatically based on the clinical variables entered into the statistical program, and the calculated NEWS was used to re-evaluate the patient’s risk assessment. For the purposes of this study, a Glasgow coma score of 14–15 was considered equivalent to A (alert) in the NEWS, and a score of <14 was considered equivalent to VPU (voice, pain, unresponsive).

The three NEWS groups (high, medium, and low) generally reflect the urgency of the patient’s needs for medical care and clinical competency of the caregivers [8]. These groups share several similarities with the Finnish A–D dispatch protocol and the three-tier EMS (Table 1). In this study, we determined the accuracy of the dispatch priority assessment as presented in Table 2. Patients with the NEWS of ≥7 (high risk) should trigger an emergency assessment by a clinical team with critical care competencies including advanced airway skills. This group was considered equal to priority A in the Finnish EMS. Patients with the NEWS of 5 or 6 (medium risk) should trigger an urgent assessment by personnel with core competencies to assess acutely ill patients, which was considered equivalent to priority B. The NEWS of <5 (low risk) warrants assessment by personnel to determine whether intensified medical care is required; this risk group was considered similar to the low urgency priorities C and D.

**Table 2:** Definition of dispatch priority accuracy

	NEWS High	NEWS Medium	NEWS Low
A priority	CORRECT	OVER-TRIAGE	OVER-TRIAGE
B priority	UNDER-TRIAGE	CORRECT	OVER-TRIAGE
C priority	UNDER-TRIAGE	UNDER-TRIAGE	CORRECT
D priority	UNDER-TRIAGE	UNDER-TRIAGE	CORRECT

Permission to perform this study was obtained from both Hospital Districts and the Office of Data Protection Ombudsman. This was a prospective registry study with an observational study design, and no clinical interventions were performed. Therefore, submission for local ethics committee approval was waived.

### Statistical analyses

Statistical analyses were performed using SPSS Statistics, version 22 [9]. Incidence rates were calculated using annual population rates from the Statistics of Finland database. When calculating the NEWS, missing values and symbols indicating normal values (e.g., ϕ, N) were considered normal (primary analysis). In addition, a complete case analysis was performed (sensitivity analysis).

Summary measurements are expressed as the mean, standard deviation, and range, unless otherwise stated. Kappa coefficient (with 95% confidence interval) was calculated as a result of reliability analysis when comparing agreement between NEWS and the priority. The Kappa coefficient measures chance-corrected proportional agreement over the whole measurement scale. The NEWS was classified as high, medium, or low, and the priority was classified as A (highest), B, or C/D. Categories C and D were combined since a kappa coefficient can only be calculated if both variables have the same number of categories. A kappa value of <0.20 indicates slight reliability, 0.21–0.40 fair reliability, 0.41–0.60 moderate reliability, 0.61–0.80 substantial reliability, and >0.80 indicates almost perfect reliability [10].

## Results

A total of 16,177 missions were carried out by the EMS during the study period, corresponding to an annual rate of 229.5 ambulance calls per 1000 inhabitants. After applying the exclusion criteria, 12,729 EMS patients were included in the present analysis. The dispatch keyword was related to illness or other medical emergency in 78.7% (10,017) of the missions, while 21.3% of the missions (2712) were trauma related. The study demographics are described in Table 3.

**Table 3:** Mission demographic data

	All	A	B	C	D
Total missions	12729 (100%)	616 (4.8%)	3193 (25.1%)	5637 (44.3%)	3283 (25.8%)
Annual mission rate per 1000 inhabitants	180.6	8.7	57.5	80.0	46.6
Mean age, years (SD)	65.2 (20.1)	61.4 (20.5)	63.7 (20.8)	64.4 (20.5)	68.7 (18.2)
Male, n (% within priority)	6275 (49.5%)	387 (63.2%)	1637 (51.5%)	2713 (48.4%)	1538 (47.0%)
00:00–08:00, n (% within priority)	2854 (22.5%)	138 (22.7%)	715 (22.4%)	1281 (22.7%)	720 (22.0%)
08:00–16:00, n (% within priority)	5291 (41.6%)	274 (45.0%)	1333 (41.8%)	2293 (40.7%)	1391 (42.4%)
16:00–24:00, n (% within priority)	4567 (35.9%)	197 (32.3%)	1141 (35,8%)	2062 (36.6%)	1167 (35.6%)
Mean EMS response time from call to arrival at the scene, minutes (SD)	13.5 (10.6)	11.1 (9.8)	10.6 (8.6)	14.0 (10.7)	16.0 (11.6)

The records of all patient charts showed symbols indicating normal values or missing data rather than exact numeric measurements for heart rate in 7.1% of the cases, systolic blood pressure in 10.8%, oxygen saturation in 11.1%, Glasgow coma scale in 12.7%, temperature in 23.3%, and respiratory rate in 56.6% of the cases. The corresponding rates of missing measurements among the urgent A–B missions were 3.1, 4.0, 5.1, 9.5, 22.7, and 41.6%, respectively. The calculated median NEWS of the study population was 1 (IQR 0–3). In 38.1% of cases, the NEWS was zero.

Figure 1 shows the distribution of the mission priorities at dispatch, as well as the NEWS classifications within each priority group. Of the priority A calls, 23.1% were categorized as high risk according to the NEWS. Among the priority B calls, 9.8% were classified as high risk, 18.9% as medium risk, and 71.3% as low risk based on the NEWS. Among the missions categorized as non-urgent C and D priorities, 87.5 and 93.5%, respectively, were classified as low risk based on the NEWS. Of the patients assigned a low-risk NEWS, 73.6% had been initially classified as non-urgent C or D priorities. Of the patients who were assigned a high-risk NEWS, 24.8 and 43.2% had initially been classified as A and B priorities, respectively, and 32.0% as low urgency priority C or D.

Comparing the dispatch assessment with the NEWS revealed that the risk assessment made by the dispatcher was correct in 67.5% of cases (Table 4). Under-triage occurred in 9.2% and over-triage in 23.4% of the cases. Among the calls classified as the highest A or B priorities, three-quarters of the calls were over-triaged. Among the calls classified as low-priority C and D missions, under-triage occurred in 12.5 and 6.5% of cases, respectively. The kappa coefficient for all missions was 0.131 (95% CI 0.109–0.152), indicating only slight reliability between these two different risk assessments.

**Table 4:** Accuracy of risk assessment, derived from the National Early Warning Score (NEWS), % of cases (number of cases)

	Correct	Under-triage	Over-triage	Total
All missions	67.5 (8589)	9.2 (1166)	23.4 (2974)	100.0 (12729)
A priority	23.1 (142)	-	76.9 (474)^a^	100.0 (616)
B priority	13.9 (445)	7.8 (248)^b^	78.3 (2500)^b^	100.0 (3193)
C priority	87.5 (4933)	12.5 (704)^c^	-	100.0 (5637)
D priority	93.5 (3069	6.5 (214)^d^	-	100.0 (3283)
Male	65.2 (4090)	10.0 (627)	24.8 (1558)	100.0 (6275)
Female	69.7 (4461)	8.4 (537)	21.9 (1399)	100.0 (6397)
16–64 years	66.0 (3615)	7.0 (383)	27.0 (1477)	100.0 (5475)
65–74 years	67.7 (1313)	11.2 (218)	21.1 (409)	100.0 (1940)
75–84 years	69.6 (2160)	10.3 (319)	20.1 (623)	100.0 (3102)
85+ years	67.9 (1501)	11.1 (246)	21.0 (465)	100.0 (2212)
00:00–08:00	67.7 (1933)	8.8 (250)	23.5 (671)	100.0 (2854)
08:00–16:00	67.4 (3567)	9.5 (501)	23.1 (1223)	100.0 (5291)
16:00–24:00	67.5 (3081)	9.1 (414)	23.5 (1072)	100.0 (4567)

^a^108 NEWS medium and 366 NEWS low missions regarded as over-triage

^b^248 NEWS high missions regarded as under-triage and 2500 NEWS low missions regarded as over-triage

^c^150 NEWS high and 554 NEWS medium missions regarded as under-triage

^d^33 NEWS high and 181 NEWS medium missions regarded as under-triage

In complete case analysis we found that every vital parameter for the NEWS calculations was measured in 4122 cases (total 32.3%; priority A 43.8%, priority B 44.2%, priority C 31.7%, priority D 19.9%). Among these, at dispatch 6.6% of the calls were prioritized as A, 34.3% as B, 43.4% as C and 15.8% as D. Based on the NEWS calculations, 9.6% of the patients were categorized as high risk, 19.8% as medium risk and 70.6% as low risk. The dispatcher’s risk assessment was correct in 54.2% of the cases (Table 5) and the Kappa coefficient was 0.098 (CI 0.068–0.128).

**Table 5:** Accuracy of risk assessment, derived from the National Early Warning Score (NEWS), % of cases (number of cases)

	Correct	Under-triage	Over-triage	Total
All missions	54.2 (2234)	18.5 (762)	27.3 (1126)	100.0 (4122)
A priority	28.1 (76)	-	71.9 (194)	100.0 (270)
B priority	21.7 (306)	12.3 (173)	66.1 (932)	100.0 (1411)
C priority	74.6 (1335)	25.4 (454)	-	100.0 (1789)
D priority	79.3 (517)	20.7 (135)	-	100.0 (652)

Complete case analysis

Risk assessment accuracy did not differ significantly with regards to gender, age, or dispatch hours (Table 3). With respect to the NEWS, the highest rate of over-triage occurred in cases of traffic accidents and among patients with a decreased level of consciousness, chest pain, stroke, or undefined disturbance of vital signs. Under-triage most commonly occurred in missions involving cardiac arrest, hypothermia, breathing difficulties, and undefined illnesses (See Additional file 2: Table S1).

## Discussion

This prospective study compared the National Early Warning Score with a criteria-based dispatch protocol for medical risk assessment in a broad EMS population. Our results showed an overall rate of 23% of over-triage and a 9% rate of under-triage with the current protocol used in Finland. About three-quarters of the calls initially categorized as the highest priorities A and B at dispatch were subsequently categorized as low risk on the scene according to the NEWS, while most missions classified as low urgency C and D priorities were also categorized as low risk based on the NEWS. To a certain extent, over-triage is necessary to ensure identification of critically ill patients from the heterogeneous population, while under-triage should be as infrequent as possible. However, it is difficult to define a reasonable level of over- or under-triage. Among trauma patients, it has been suggested that rates of 1–5% under-triage and 25–50% over-triage are acceptable [10].

Our present findings showed that almost a quarter of all missions were over-triaged. Only 23% of the highest priority missions were still considered high priority in the NEWS risk assessment made on the scene. Similarly, a Swedish study found a 27% correspondence when comparing the first dispatch priority made by EMCC with a second priority assessment made by the ambulance crew on the scene using the Swedish criteria-based triage protocol [4]. A Norwegian study demonstrated that more than 70% of all highest priority missions were found to be non-life-threatening situations [11]. These high rates of overestimation lead to inappropriate use of limited EMS resources. If EMS units are occupied with low-risk patients, a simultaneous high-risk patient may be reached with long delays. Unnecessary light-and-siren calls also increase the risk of traffic accidents, endangering EMS personnel as well as other road users [1].

In our present study more than 9% of EMS missions were considered under-triaged. Of concern is that one-third of the patients with a high medical risk according to the NEWS on the scene were initially classified as low urgency C or D priorities at dispatch. This high proportion of under-triage could potentially have an irrecoverable impact on the high-risk patients’ morbidity or even survival.

In this material, 29.9% of the calls were initially classified as the highest priority A and B calls. Similar priority distributions have been reported in previous studies of the Finnish EMS system, including 32.7% A and B calls in the Helsinki metropolitan area, [12] and 34.5% in Southern-Finland urban area [13]. Denmark also uses a criteria-based dispatch protocol with priorities from A to D, but the priority definitions differ considerably from the Finnish system. A study from the Central Region of Denmark showed a 51.4% prevalence of high-priority missions. The Danish study also demonstrated a significantly lower annual rate of ambulance missions: 32.2 per 1000 inhabitants compared to 229.5 per 1000 inhabitants in the present study [14]. It is difficult to explain these striking differences between the two countries, but they may be due to differences in the dispatch protocols, EMCC personnel education as well as cultural differences related to contacting the EMCC.

A previous Canadian study found that 16 of the 32 keywords had a sensitivity of less than 50% for detecting high-acuity patients [3]. Similarly, in our present series, comparison with the NEWS revealed that risk assessment was correct in less than 50% of the calls that were categorized using 14 out of the 40 dispatch keywords (Additional file 2: Table S1). There are several explanations why some keywords in the Finnish national dispatch protocol are associated with categorization that over- or under-estimates the anticipated risk at dispatch in comparison to the NEWS. First, our results falsely indicate an underestimation of cardiac arrest patients. Due to their poor prognosis, patients found in unwitnessed cardiac arrest are categorized as priority B and not priority A by the Finnish dispatch protocol, while the NEWS considers these to be high-risk patients. Second, our findings may falsely indicate an overestimation of chest pain and stroke patients. Without disturbances of vital functions, these patients would be allocated a low NEWS, but patients with acute ischemic ECG findings or neurological hemiplegic symptoms require urgent medical interventions, and therefore chest pain and stroke symptoms are classified as high-risk missions at dispatch.

Our present results indicated that the current Finnish dispatch protocol is not optimal, even though it is an automated system that relies on protocol-based call taking. Earlier studies have promoted these features because they improve ambulance utilization [15, 16]. In 2006, Finland implemented a nationwide standardization of the EMCCs. However, it has been previously reported that this change did not improve the accuracy of risk assessment as evaluated by correct recognition of cardiac arrest, stroke, and STEMI patients in one of the regional dispatch centres [17]. This fact further highlights the need for focused studies to re-evaluate the current protocol. The dispatch protocol must be made more sensitive to decrease under-triage to the acceptable rate of less than 5% and more specific to reduce over-triage of the highest priority A and B calls. The effectiveness of the protocol affects individual patients, and has major impacts from a financial point of view and with regard to resource deployment of the EMSs.

In the present study, we compared the risk assessment made using the Finnish criteria-based dispatch protocol with the NEWS. Since dispatch protocols differ among countries, our results are not fully generalizable to countries using different criteria-based protocols. Despite the standardized use of the Finnish protocol, this protocol has not been validated. There remains a need for studies comparing the efficacies and accuracies of different national protocols, to promote development of a more accurate and validated protocol. The dispatch protocol needs to be re-evaluated by analysing specific dispatch criteria for selected key words to identify possible information gaps. Studies investigating short- and long-term patient outcomes are also needed. To enable high-quality research of the topic in the future, there is foremost a need to create a Finnish national EMS database, including EMCC data system combined with the national EMS patient data archive.

The strengths of this study include its prospective design and the inclusion of EMS patients from all priority categories. This study also has some evident limitations. First, missing values and symbols indicating normal (e.g., ϕ) were considered normal, which may have had an impact on the calculated NEWS. The complete case analysis without missing values suggests the rate of over-triage at the priority A and B missions to be lower and the rate of under-triage at the priority C and D missions to be higher (Table 5). However, in this sensitivity analysis physiological measurements were performed more comprehensively for high-priority A and B missions indicating that the patients who are assessed sicker by EMS personnel are evaluated in more detail. This is also reflected in daily practise, where it does not seem suitable to measure all vital parameters in all patients—for example, in cases of psychiatric disorders or minor injuries. Therefore, considering missing values to be normal is reasonable to some extent. Second, NEWS is affected by underlying chronic diseases or chronic abnormalities in the physiological parameters — for example, oxygen saturation and level of consciousness — and thus the NEWS may overestimate the true medical risk among these patients. As discussed earlier, some types of medically high-risk patients do not receive a high NEWS. For these patients, the healthcare professional’s evaluation will override the NEWS. Third, dispatcher compliance with the current dispatch protocol is not documented; therefore, the findings could be skewed by dispatchers’ subjective decisions. Finally, it must be admitted that comparing dispatch accuracy with NEWS is unfair to the dispatcher because NEWS as a physiological scoring system can never provide the dispatcher with the information obtained on the scene. Despite this, we feel that NEWS can be a valuable tool to develop dispatching criteria.

## Conclusion

The present results show that the current Finnish medical dispatch protocol is suboptimal and needs to be further developed. A substantial proportion of EMS missions assessed as highest priority were categorized as lower risk according to the NEWS determined at the scene, indicating over-triage with the protocol. On the other hand, only a quarter of the high risk NEWS patients were classified as the highest priority at dispatch, indicating considerable under-triage with the protocol.

## Additional files

Additional file 1: NEWS usage; calculation of NEW-score and definition of the clinical risk. (DOCX 137 kb)
Additional file 2: Table S1. Distribution of mission codes, priorities, National Early Warning Scores (NEWS), and accuracy of the risk assessment derived from the NEWS. (DOCX 146 kb)

## Abbreviations

ECG: Electrocardiography
EMCC: Emergency Medical Communication Centre
EMS: Emergency medical service
NEWS: National early warning score
STEMI: ST-elevation myocardial infarct
