# Supplementary material for: Medical priority dispatch codes—comparison with National Early Warning Score
Source: Scand J Trauma Resusc Emerg Med. 2016 Dec 3;24:142. doi: 10.1186/s13049-016-0336-y (PMC5135813; doi:10.1186/s13049-016-0336-y)

Additional file 1.

NEWS usage; calculation of NEW-score and definition of the clinical risk. Charts adapted from *Royal College of Physicians. National Early Warning Score (NEWS): Standardizing the assessment of acute-illness severity in the NHS. Report of a working party. London: 2012.*

Chart 1: National Early Warning Score (NEWS)

| Physiological parameters | 3 | 2 | 1 | 0 | 1 | 2 | 3 |
| --- | --- | --- | --- | --- | --- | --- | --- |
| Respiration Rate | ≤8 |  | 9 - 11 | 12 - 20 |  | 21 -24 | ≥25 |
| Oxygen Saturations | ≤91 | 92 - 93 | 94 - 95 | ≥96 |  |  |  |
| Any Supplemental Oxygen |  | Yes |  | No |  |  |  |
| Temperature | ≤35.0 |  | 35.1 – 36.0 | 36.1 – 38.0 | 38.1 – 39.0 | ≥39.1 |  |
| Systolic Bloodpressure | ≤90 | 91 - 100 | 101 -110 | 111 - 219 |  |  | ≥220 |
| Heart Rate | ≤40 |  | 41 - 50 | 51 - 90 | 91 - 110 | 111 - 130 | ≥131 |
| Level of Consciousness |  |  |  | A |  |  | V, P, or U |

Chart 2: NEWS thresholds and triggers

| NEW scores | Clinical risk |
| --- | --- |
| 0 | Low |
| Aggregate 1-4 |  |
| Individual parameter scoring 3 | Medium |
| Aggregate 5-6 |  |
| Aggregate 7 or more | High |


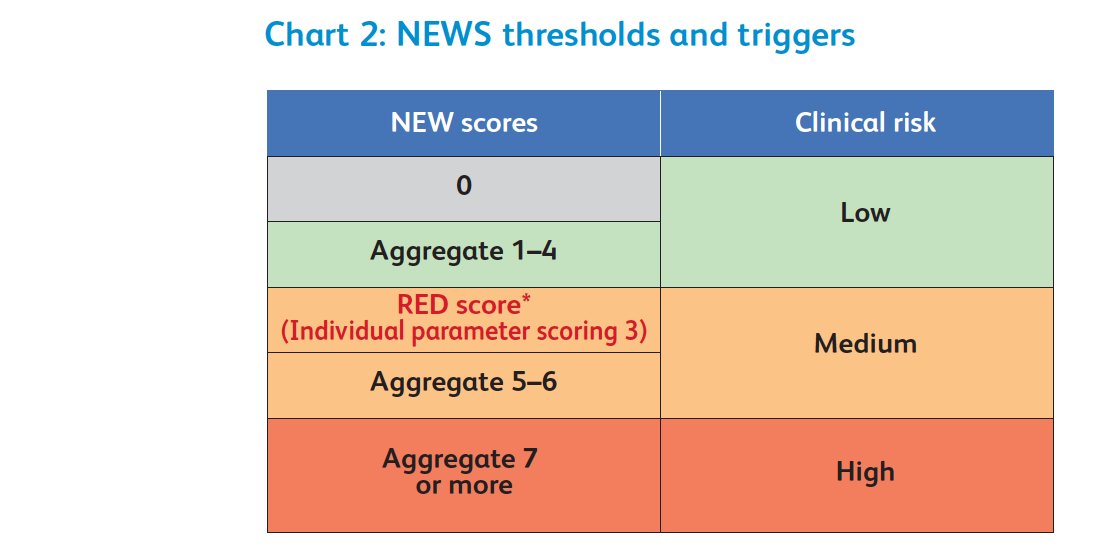

Supplement: Additional file 1: — NEWS usage; calculation of NEW-score and definition of the clinical risk. (DOCX 137 kb) [file 13049_2016_336_MOESM1_ESM.docx]
