# Supplementary material for: Medical priority dispatch codes—comparison with National Early Warning Score
Source: Scand J Trauma Resusc Emerg Med. 2016 Dec 3;24:142. doi: 10.1186/s13049-016-0336-y (PMC5135813; doi:10.1186/s13049-016-0336-y)
Supplement: Additional file 2: Table S1. — Distribution of mission codes, priorities, National Early Warning Scores (NEWS), and accuracy of the risk assessment derived from the NEWS. (DOCX 146 kb) [file 13049_2016_336_MOESM2_ESM.docx]

| Mission codes |  | A-priority | | B-priority | | C-priority | | D-priority | | Correct | Under-triage | Over-triage |
| --- | --- | --- | --- | --- | --- | --- | --- | --- | --- | --- | --- | --- |
|  | N (% of total calls) | N (% within priority | Median NEW-score (IQR) | N (% within priority | Median NEW-score (IQR) | N (% within priority | Median NEW-score (IQR) | N (% within priority | Median NEW-score (IQR) | % (n) | % (n) | % (n) |
| Medical missions |  |  |  |  |  |  |  |  |  |  |  |  |
| 700 Cardiac arrest | 103 (0.8) | 60 (9.7) | 16 (15-18) | 43 (1.3) | 15 (15-18) | - | - | - | - | 42.7 (44) | 38.8 (40) | 18.4 (19) |
| 702 Unconsciousness | 136 (1.1) | 71 (11.5) | 4 (2-9) | 65 (2.0) | 4 (1-5) | - | - | - | - | 36.0 (49) | 8.1 (11) | 55.9 (76) |
| 703 Breathing difficulties (no external cause) | 972 (7.6) | 49 (8.0) | 7 (4-10) | 318 (10.0) | 3 (1-6) | 605 (10.7) | 3 (1-5) | - | - | 53.4 (519) | 26.3 (256) | 20.3 (197) |
| 704 Chest pain | 1345 (10.6) | 174 (28.2) | 1 (0-3) | 820 (25.7) | 1 (0-2) | 351 (6.2) | 1 (0-2) | - | - | 29.1 (391) | 3.1 (42) | 67.8 (912) |
| 705 Undefined disturbances of vital functions | 1584 (12.4) | 61 (9.9) | 2 (1-4) | 493 (15.4) | 2 (1-3) | 1030 (18.3) | 1 (0-2) | - | - | 61.6 (975) | 11.2 (178) | 27.2 (431) |
| 706 Stroke | 360 (2.8) | - | - | 262 (8.2) | 1 (0.3) | 98 (1.7) | 1 (0.2) | - | - | 37.2 (134) | 4.4 (16) | 58.3 (210) |
| 711 Airway obstruction (foreign object) | 20 (0.2) | 8 (1.3) | 3 (1.5-5) | 12 (0.4) | 1 (0-2) | - | - | - | - | 15.0 (3) | 5.0 (1) | 80.0 (16) |
| 713 Hanging | 5 (0.0) | 2 (0.3) | 5.5 (4-7) | 3 (0.1) | 2 (1-10) | - | - | - | - | 20.0 (1) | 20.0 (1) | 60.0 (3) |
| 714 Drowning | 6 (0.0) | 5 (0.8) | 6 (0-15) | 1 (0.0) | * (*-*) | - | - | - | - | 33.3 (2) | 16.7 (1) | 50.0 (3) |
| 741 Falling from height | 16 (0.1) | 5 (0.8) | 2 (0-3) | 11 (0.3) | 1 (0-3) | - | - | - | - | 6.3 (1) | 6.3 (1) | 87.5 (14) |
| 742 Wound | 140 (1.1) | 1 (0.2) | * (*-*) | 18 (0.6) | 1 (0-3) | 72 (1.3) | 0 (0-1) | 49 (1.5) | 0 (0-1) | 85.0 (119) | 3.6 (5) | 11.4 (16) |
| 745 Falling, stumbling (not from height) | 1525 (12.0) | 16 (2.6) | 1.5(1-2.5) | 149 (4.7) | 1 (0-2) | 810 (14.4) | 1 (0-2) | 550 (16.8) | 0 (0-1) | 84.4 (1287) | 6.1 (93) | 9.5 (145) |
| 746 Blow, hit | 25 (0.2) | 1 (0.2) | * (*-*) | 7 (0.2) | 1 (0-1.5) | 17 (0.3) | 0 (0-1) | - | - | 72.0 (18) | - | 28.0 (7) |
| 747 Other trauma (not MVA) | 60 (0.5) | 6 (1.0) | 0.5 (0-1) | 24 (0.8) | 0 (0-1) | 30 (0.5) | 0 (0-1) | - | - | 48.3 (29) | 1.7 (1) | 50.0 (30) |
| 751 Inhalation of toxic gas | 15 (0.1) | 3 (0.5) | 2 (0.5-6.5) | 3 (0.1) | 3 (2.5-3) | 9 (0.2) | 1 (0-2) | - | - | 73.3 (11) | - | 26.7 (4) |
| 752 Substance abuse, Overdose | 286 (2.2) | 11 (1.8) | 2 (0.5-6) | 114 (3.6) | 2 (1-4) | 160 (2.8) | 2 (1-3) | 1 (0.0) | * (*-*) | 55.9 (160) | 13.3 (38) | 30.8 (88) |
| 753 Electrocution | 4 (0.0) | - | - | 2 (0.1) | 2 (0.4) | 2 (0.0) | * (*-*) | - | - | 50.0 (2) | - | 50.0 (2) |
| 754 Burns | 10 (0.1) | - | - | 6 (0.2) | 1 (0-2) | 4 (0.1) | 0.5 (0-1) | - | - | 40.0 (4) | - | 60.0 (6) |
| 755 Heat injury | 4 (0.0) | - | - | 3 (0.1) | 2 (1.5-3) | 1 (0.0) | * (*-*) | - | - | 25.0 (1) | - | 75.0 (3) |
| 756 Cold injury | 44 (0.3) | 1 (0.2) | * (*-*) | 11 (0.3) | 3 (1-3.5) | 32 (0.6) | 2 (1-4) | - | - | 59.1 (26) | 31.8 (14) | 9.1 (4) |
| 761 Hematemesis | 46 (0.4) | - | - | 15 (0.5) | 2 (0.5-7) | 22 (0.4) | 0.5 (0-2) | 9 (0.3) | 1 (0-2) | 60.9 (28) | 17.4 (8) | 21.7 (10) |
| 762 Vaginal or rectal bleeding | 110 (0.9) | 1 (0.2) | * (*-*) | 16 (0.5) | 1.5 (0-2) | 65 (1.2) | 1 (0-2) | 28 (0.9) | 0 (0-2) | 79.1 (87) | 5.5 (6) | 15.5 (17) |
| 763 Nose bleeding | 142 (1.1) | - | - | 5 (0.2) | 1 (1-1) | 104 (1.8) | 1 (0-1) | 33 (1.0) | 1 (0-1) | 95.1 (135) | 1.4 (2) | 3.5 (5) |
| 764 Bleeding from surgical or varicose ulcer | 40 (0.3) | - | - | 2 (0.1) | 0.5 (0-1) | 25 (0.4) | 1 (0-1) | 13 (0.4) | 0 (0-0) | 95.0 (38) | - | 5.0 (2) |
| 770 Undefined illness, sudden | 88 (0.7) | - | - | 2 (0.1) | 1 (0-3) | 25 (0.4) | 1 (0-3) | 13 (0.4) | - | 8.0 (7) | 10.2 (9) | 81.8 (72) |
| 771 Diabetic problem | 172 (1.6) | 19 (3.1) | 3 (2-4.5) | 52 (1.6) | 1 (1-3) | 101 (1.8) | 1 (0-3) | - | - | 59.3 (102) | 8.1 (14) | 32.6 (56) |
| 772 Convulsions | 270 (2.1) | 38 (6.2) | 5 (2-7) | 153 (4.8) | 2 (1-4) | 78 (1.4) | 1 (0-2) | 1 (0.0) | * (*-*) | 45.2 (122) | 8.1 (22) | 46.7 (126) |
| 773 Allergic reaction | 56 (0.4) | 3 (0.5) | 1 (0.5-2.5) | 27 (0.8) | 1 (0-2.5) | 26 (0.5) | 1 (0-1) | - | - | 51.8 (29) | 1.8 (1) | 46.4 (26) |
| 774 Undefined illness, chronic | 1905 (15.0) | 1 (0.2) | * (*-*) | - | - | 740 (13.1) | 1 (0-3) | 1164 (35.5) | 1 (0-3) | 86.0 (1639) | 14.0 (266) | - |
| 775 Vomiting, diarrhea | 415 (3.3) | - | - | - | - | 164 (2.9) | 1 (0-2) | 251 (7.6) | 0 (0-2) | 93.7 (389) | 6.3 (26) | - |
| 781 Abdominal pain | 572 (4.5) | 3 (0.5) | 0 (0-1.5) | 86 (2.7) | 0 (0-1.5) | 333 (5.9) | 1 (0-2) | 150 (4.6) | 0 (0-1) | 82.0 (469) | 3.7 (21) | 14.3 (82) |
| 782 Headache | 205 (1.6) | - | - | 21 (0.7) | 1 (0-1) | 117 (2.1) | 0 (0-1) | 67 (2.0) | 0 (0-1) | 87.8 (180) | 2.4 (5) | 9.8 (20) |
| 783 Back-, limb- or trunk pain | 974 (7.7) | - | - | 10 (0.3) | 1 (0-1) | 304 (5.4) | 1 (0-1) | 660 (20.1) | 0 (0-1) | 94.5 (920) | 4.5 (44) | 1.0 (10) |
| 785 Mental disorder | 360 (2.8) | - | - | - | - | 60 (1.1) | 0 (0-1) | 300 (9.1) | 0 (0-1) | 96.7 (348) | 3.3 (12) | - |
| 790 Urgent dispatch, call still in process | 101 (0.8) | 1 (0.2) | * (*-*) | 98 (3.1) | 3 (1-5) | 2 (0.0) | * (*-*) |  |  | 26.7 (27) | 17.8 (18) | 55.4 (56) |
| 791 Pregnancy or birth | 57 (0.4) | 3 (0.5) | 0 (0-1) | 24 (0.8) | 0.5 (0-1) | 24 (0.4) | 1 (0-1) | 6 (0.2) | 0 (0-1) | 52.6 (30) | - | 46.4 (27) |
| 795 Stand-by for EMS-mission | 4 (0.0) | - | - | - | - | 4 (0.1) | 4 (1-12) | - | - | 50.0 (2) | 50.0 (2) | - |
| Fire and rescue missions |  |  |  |  |  |  |  |  |  |  |  |  |
| 2** Traffic accidents | 361 (2.8) | 50 (8.1) | 1 (0-2) | 190 (6.0) | 1 (0-2) | 121 (2.1) | 0 (0-1) | - | - | 35.7 (129) | 2.2 (8) | 62.0 (224) |
| 402 House fire | 2 (0.0) | - | - | - | - | 2 (0.0) | 1 (0-2) | - | - | 100.0 (2) | - | - |
| Police missions |  |  |  |  |  |  |  |  |  |  |  |  |
| 031 Gunshot | 8 (0.1) | 4 (0.6) | 16.5 (15-18) | 4 (0.1) | 1 (0-10) | - | - | - | - | 50.0 (4) | 12.5 (1) | 37.5 (3) |
| 032 Stabbing | 22 (0.2) | 12 (1.9) | 1 (0-1.5) | 8 (0.3) | 0.5 (0-1.5) | 2 (0.0) | 1 (0-2) | - | - | 13.6 (3) | - | 86.4 (19) |
| 033 Assault | 159 (1.2) | 6 (1.0) | 1.5 (1-5) | 30 (0.9) | 1 (0-2) | 122 (2.2) | 0 (0-1) | 1 (0.0) | * (*-*) | 77.4 (123) | 1.9 (3) | 20.8 (33) |

**Additional file 2: Table S1. Distribution of mission codes, priorities, National Early Warning Scores (NEWS), and accuracy of the risk assessment derived from the NEWS.**
